# The Management of Labor and the Lying-in Period

**Published:** 1885-12

**Authors:** 


**The Management of Labor and of the Lying-in Period.** A Guide for the young practitioner, by HENRY G. LANDIS, A. M., M. D., Professor of Obstetrics, Starling Medical College, etc. Philadelphia: Lea Brothers & Co. 1885.

The aim of this book, as the author states, is "to serve as a guide to practice, divested of all superfluous and irrelevant details." The neat, concise and instructive book which this is, shows success, which could only be attained by a teacher of large experience. It does not pretend to be a treatise on obstetrics, and, therefore, takes for granted an acquaintance with the anatomy and physiology of the parts involved, and of the mechanism of normal labor. Dr. Landis' opinions and directions are sound and practical, the language is clear and forcible, and, though the book is intended for students, it will repay perusal by those who have had experience. The chapter on forceps and their use is an admirable one, and those who read it will be glad to obtain the author's treatise, which, on its appearance, we commended, entitled " How to use the Forceps."

